# Use of service data to inform pediatric HIV-free survival following prevention of mother-to-child transmission programs in rural Malawi

**DOI:** 10.1186/1471-2458-12-405

**Published:** 2012-06-06

**Authors:** Justin Mandala, Tiwonge Moyo, Kwasi Torpey, Mark Weaver, Chiho Suzuki, Rebecca Dirks, Chika Hayashi

**Affiliations:** 1 FHI 360, Washington, DC, USA; 2 FHI 360, Lilongwe, Malawi; 3 FHI 360, Abuja, Nigeria; 4 University of North Carolina, Division of General Medicine and Clinical Epidemiology, Chapel Hill, NC, USA; 5 UNICEF, New York, NY, USA; 6 World Health Organization, Geneva, Switzerland

## Abstract

**Background:**

Recent years have seen rapid and significant progress in science and implementation of programs to prevent mother-to-child transmission of HIV. Programs that support PMTCT routinely monitor service provision but very few have measured their effectiveness. The objective of the study was to use service data to inform HIV-free survival among HIV exposed children that received antiretroviral drugs to prevent mother-to-child transmission (PMTCT) of HIV. The study was conducted in two rural districts in Malawi with support from FHI 360.

**Methods:**

A descriptive observational study of PMTCT outcomes was conducted between June 2005 and June 2009. The dataset included patient-level data of all pregnant women 1) that tested HIV-positive, 2) that were dispensed with antiretroviral prophylaxis, and 3) whose addresses were available for home visits. The data were matched to each woman’s corresponding antenatal clinic data from home visit registers.

**Results:**

Out of 438 children whose home addresses were available, 33 (8%) were lost to follow-up, 35 (8%) were alive but not tested for HIV by the time home visit was conducted, and 52 (12%) were confirmed deceased. A total of 318 children were alive at the time of the home visit and had an HIV antibody test done at median age 15 months. The resulting estimated 24-month probability of HIV-free survival over all children was 78%. Among children who did not receive nevirapine, the estimated 24-month probability of HIV-free survival was 61%, and among those who did receive NVP syrup the estimate was 82%.

**Conclusions:**

When mothers and newborns received nevirapine, the estimated 24-month probability of HIV-free survival among children was high at 82% (CI: 54% to 99%). However this conclusion should be interpreted cautiously 1) due to the wide confidence interval; and 2) because the confidence interval range includes 55%, which is the natural HIV-free survival rate in the absence of a PMTCT intervention. This analysis highlighted the need of quality data and well-structured home visits to assess PMTCT effectiveness.

## Background

Mother-to-child transmission of HIV (MTCT) accounts for an estimated 1,000 pediatric HIV infections every day, the majority of which occur in sub-Saharan Africa [1]. In this part of the world, the combination of high HIV prevalence, high birth rates and under-resourced health systems have resulted in a heavy toll on children’s health outcomes.

Recent years have seen rapid and significant progress in science and implementation of programs to prevent MTCT. Proven interventions for prevention of mother-to-child transmission (PMTCT) are available and are being implemented globally, including resource-constrained settings [2-4]. Programs that support PMTCT routinely monitor service provision but very few have measured their effectiveness [5,6].

Data on effectiveness of PMTCT programs are crucial to not only gauge if programmatic and operational targets are being reached, but also to continually refine provision of PMTCT services in order to control the pediatric HIV epidemic.

The objective of this study was to use service data to estimate HIV-free survival among children born to HIV-positive mothers that received antiretroviral (ARV) drugs in the context of PMTCT programs in two rural districts in Malawi between June 2005 and June 2009.

### Program description

Malawi has one of the highest HIV seroprevalence rates among sub-Saharan countries; in 2004 a population based survey estimated that 12.7% of adults between 15 and 49 years old were living with HIV [1,7]. In 2005, HIV seroprevalence among pregnant women was estimated to be 27% in urban areas [8]. An estimated 100,000 children below the age of 15 live with HIV and one-fifth of the 99.1 deaths per 1,000 children under 15 are attributable to HIV [1,9]. PMTCT coverage remains a significant problem in Malawi; in 2009, only an estimated 58% of HIV-positive pregnant women with HIV infection and 41% of HIV-exposed infants were provided with ARVs to prevent MTCT [10].

Between 2005 and 2009, in partnership with the Ministry of Health of Malawi, FHI 360 supported a program to mitigate the impact of HIV on children in Chikwawa and Dowa, two rural districts. Chikwawa and Dowa have an estimated HIV seroprevalence of 11% and a population of 435,000 and 560,000 inhabitants respectively [11,12]. A specific objective of the program was to scale up PMTCT programs in 15 health centers (eight in Chikwawa and seven in Dowa). FHI 360 support consisted of training and mentoring on PMTCT, commodities procurement, data collection, reporting and quality assurance as well as community mobilization.

FHI 360 supported the Ministry of Health to train and re-train all relevant health personnel on national PMTCT guidelines and the importance that they be systematically followed. During the training, in an effort to strengthen the follow-up component of PMTCT and improve documentation during antenatal visits and home visits, specific emphasis was given to proper record keeping and report writing using the existing national tools. The PMTCT program spanned the cascade of services, including HIV counseling and testing of pregnant women, provision of ARV prophylaxis among those identified as HIV positive, provision of infant ARV prophylaxis, provision of infant HIV testing, and follow-up home visits. All HIV-positive mothers and their HIV-exposed children were referred for comprehensive HIV clinical care including lifelong antiretroviral treatment (ART) as needed. At the time of implementation, single dose nevirapine (sdNVP) was the backbone of PMTCT programs in rural Malawi; complex ARV regimens beyond sdNVP were not being implemented.

PMTCT related clinical activities-such as testing and counseling for HIV, provision of sdNVP to HIV-positive mothers, and provision of nevirapine (NVP) syrup to the newborn were carried out by nurses and midwives.

Outside of the health centers, community health workers conducted monthly home visits to HIV-positive mothers that received ARVs and to their HIV-exposed children between zero and 24 months postnatal. Home visits were routine practice conducted by community health workers. During home visits, psychosocial support, adherence counseling for cotrimoxizole preventative therapy and ARVs, and HIV care were provided. Additionally an assessment of the HIV and living status of the infants was conducted. For this study, HIV status was recorded from the most recent test; there was no documentation on the number or outcomes of HIV tests performed previously. Lastly, the health passports of the mothers were reviewed to confirm their attendance at follow-up appointments and make new appointments if necessary. Health passports are individual books provided to each client when first attending a health facility.

Data from both health facilities and community based activities were routinely collected. Key indicators for PMTCT were integrated into registers from antenatal clinic (ANC) and registers from other complimentary services such as HIV counseling and testing, family planning, and sexually transmitted infections.

At the time of program implementation, the national policy of Malawi on HIV diagnosis included three rapid tests: Determine HIV 1/2 for screening, Uni-Gold HIV test for confirmation and SD Bioline HIV1/2 3.0 as a tiebreaker. The first two tests were run in parallel until May 2008 when the national policy changed to serial testing. The national policy recommended new HIV rapid test kits in 2008, but by the end of the program supported by FHI 360, the change was not yet operational in rural sites.

In practice, HIV-exposed children were tested for HIV at different ages as a result of several key challenges. First, it was not uncommon for mothers to not return to the health facility on their appointment date to have their child examined and tested. Next, the follow-up home visits were not always scheduled at consistent time intervals, due to shortages of health care workers and high client volumes. Lastly, there were a number of parents who relocated due to employment-especially in Chikwawa which is a sugarcane growing area. Large sugar estates attract people seeking seasonal employment.

## Methods

### Design

This is a descriptive observational study of PMTCT activities.

### Site selection

The 13 PMTCT sites included in the analysis were among the fifteen supported by FHI 360 in the Chikwawa and Dowa districts. Two of the fifteen FHI 360-supported facilities were not included because they did not offer follow-up of mother-baby pairs beyond ANC.

### Data collection and entry

Patient-level data collected from the ANC register included information on all pregnant women 1) that tested HIV-positive, 2) that were dispensed with ARV prophylaxis, and 3) whose home visit information was available. The data were matched to each woman’s corresponding ANC data from the home visit register. For the purpose of this study the following data were extracted from the ANC register: 1) age of the mother at the first ANC visit, 2) parity, 3) distance in kilometers (km) from her house to the health center, and 4) whether the woman’s HIV-positive status was disclosed to her partner. From the home visit register, additional data were extracted: 1) mother’s living status, 2) mother’s access to HIV clinical care, 3) whether postnatal NVP was given to the infant at birth, 4) child’s living status, 5) infant feeding method, and 6) child’s HIV status.

The accuracy of the data collected both at health facilities and during home visits was monitored throughout the implementation of the PMTCT programs by FHI 360’s monitoring and evaluation team. These data were double-entered into a Microsoft Excel database version 2003 (2).

### Data analysis

The outcome considered for this analysis is HIV-free survival until the age of 24 months. HIV-free survival is defined as being alive and confirmed HIV-uninfected at the time of home visits.

Because the exposed infant’s HIV status was first assessed and documented between the ages of six weeks and 24 months after birth, it was not possible to know precisely when HIV infection occurred. For example, an infant who was first tested for HIV at age six months and was found to be positive could have become infected any time between birth and six months. Similarly, an infant who was known to have died by six months of age may have become infected prior to death. Thus, nonparametric methods for interval censored data [13] (similar to Kaplan-Meier methods) were used to estimate, along with 95% confidence intervals, the cumulative probability of HIV-free survival at 24 months postnatal.

In univariate and multivariate models, hazard ratios were estimated, along with 95% confidence intervals, using parametric Weibull proportional hazards models, which account for interval censored data, to explore the associations between HIV-free survival and 1) sex of the child, 2) mother’s living status, 3) duration of time an infant was breastfed (less than six months vs. over six months), 4) whether the child received NVP, and 5) whether the mother received (lifelong) ART. Data analysis was performed using SAS, Version 9.2 (SAS Institute, Cary, NC, USA).

### Ethical approval

This data review was submitted to the FHI 360′s Protection of Human Subjects Committee. It was determined that this study does not constitute human subject research as defined under DHHS regulation 45 CFR 46.102 (d) & (f) and therefore IRB review and approval were not required.

## Results

### Data overview

Between June 2005 and June 2009, a total of 40,691 pregnant women were registered in FHI 360 supported antenatal clinics; 40,139 were counseled and tested for HIV (98.6% uptake) and 2,408 women were found to be HIV-positive (6.0% HIV prevalence). Of those who tested HIV positive, 1,851 were dispensed with sdNVP (76.9% uptake of ARV prophylaxis among mothers) and 1,353 of newborns to HIV-positive mothers received NVP syrup (56.2% uptake of infant ARV prophylaxis). The uptake of PMTCT interventions was higher in FHI 360 supported sites than the national average. The drop-out in number of women and their HIV-exposed neonates receiving ARV is due to the low proportion (56%) of women completing the four antenatal visits and the low proportion (50%) of institutional delivery observed in rural Malawi [10]. Not all pregnant women tested HIV-positive came back to be dispensed with NVP tablets and even fewer brought their neonates to be provided with NVP syrup.

In this analysis, 438 HIV-positive pregnant women were registered in antenatal books, had received sdNVP, and had received follow up home visits. These 438 patients represent 23.6% of women that tested HIV positive and were dispensed with ARVs; the remaining (76.4%) could not be followed because their home addresses were missing from the registers.

The mothers’ median age was 28.0 years; they lived a median of 5 km from the antenatal clinic, and had a median parity of 3. Overall, 99% of HIV-exposed children had ever been breastfed, 62% of those for over six months. The profile of mothers who were lost to follow-up (LTFU) was comparable to the profile of those who were able to be tracked through home visits (Table 1).

**Table 1:** Profile of mothers included in the review

	**Among mothers not LTFU**						**Among mothers LTFU**					
	*N*	Mean	Std	Median	Min	Max	*N*	Mean	Std	Median	Min	Max
Mothers’ age	*369*	27.9	5.6	28	12	44	*30*	28.7	5.8	28	18	41
Mothers parity	*372*	3.3	1.9	3	1	11	*23*	3.4	1.8	4	1	7
Kms to ANC*	*382*	7.8	9.5	5	0.1	50.0	*3*	2.5	3.0	1	0.5	6.0

* Distance in kilometers to the antenatal clinic.

**Figure 1:**
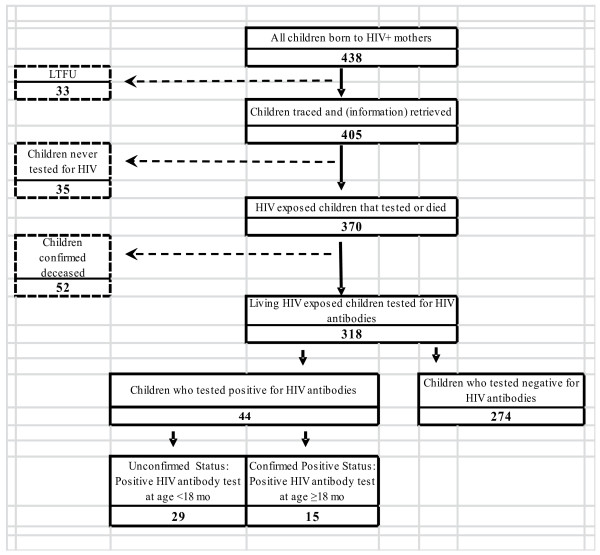
HIV-free survival flow chart.

As shown in Figure 1, of the 438 children whose mothers received sdNVP and provided their addresses, 33 (8%) were considered LTFU, i.e. after delivery, community health workers could not locate the mothers or their children despite availability of their home address in the register. Furthermore, 35 infants (8%) were alive but were never tested for HIV by the time home visit was conducted and 52 (12%) were confirmed deceased. A total of 318 children were alive at the time of the home visit and had an HIV antibody test conducted prior to the time of home visit. The median age of the children was 15 months, with a range of six weeks to 33.8 months. Of those 318 children, 274 tested negative and 44 tested positive for HIV antibodies. Among the 44 with a positive result, 15 were tested at age 18 months or older, and therefore confirmed to be infected with HIV. However the HIV status of the remaining 29 infants, who were tested prior to age 18 months, could not be definitely established per WHO guidelines on the diagnosis of HIV in children [14]. Note that in this analysis, infants who tested positive for HIV antibodies prior to age 18 months were categorized as HIV-infected in the determination of HIV-free survival.

### Overall HIV-free survival

Of the 438 HIV-exposed children whose mothers received sdNVP and provided their address, 349 were included in the analysis. A total of 89 children were excluded from analysis due to missing data.

The resulting estimated 24-month probability of HIV-free survival among all children was 78%, with 95% confidence interval (CI) 41% to 99%. Among children who did not receive NVP syrup, the estimated 24-month probability of HIV-free survival was 61% (CI: 21% to 99%), and among those who did receive NVP syrup the estimate was 82% (CI: 54% to 99%).

### Factors associated with HIV-free survival

The results from the univariate and multivariate proportional hazards models for interval censored data are shown in Table 2. Among the factors explored, only “children who did receive NVP” was significantly associated with HIV-free survival; the estimated hazard ratio was 2.77 (CI: 1.61 to 4.78). Being female child was associated with lower HIV-free survival; however, the significance was borderline-HR 1.65 (CI: 0.99 to 2.75).

**Table 2:** Estimated* hazard ration (HR) for selected factors associated with HIV-free survival

**Factors**	**Univariate analysis**			**Multivariate analysis**		
	HR	95% CI	*P*-value	HR	95% CI	*P*-value
Child is female	1.54	(0.96, 2.46)	0.071	1.65	(0.99, 2.75)	0.053
Mother not alive	2.09	(0.51, 8.62)	0.309	1.04	(0.14, 7.85)	0.968
Breastfed < 6 months	0.95	(0.13, 7.02)	0.961	1.63	(0.21, 12.94)	0.642
Breastfed ≥6 months	0.79	(0.11, 5.75)	0.813	1.49	(0.19, 11.90)	0.705
Child did not receive NVP	2.76	(1.69, 4.49)	< 0.001	2.77	(1.61, 4.78)	< 0.001
Mother not on ART when breastfeeding	0.89	(0.55, 1.43)	0.620	0.71	(0.42, 1.21)	0.207

* From a parametric Weibull proportional hazards model.

## Discussion

This study has demonstrated the usefulness and limitations of using service data to inform HIV-free survival as a result of PMTCT programs. It measured not only the proportion of infants that were not HIV-infected, but also the ultimate outcome of PMTCT intervention: a living child free of HIV infection. A key contribution of this study has also been to describe the limitations of analyzing service data to measure outcomes of PMTCT programs.

Analysis of service data from the sdNVP based PMTCT interventions in rural Malawi provides some limited indication of reduced risk of MTCT [15,16]. In this context of high breastfeeding rates, without any intervention, HIV-free survival at 24 months postnatal could be as low as 55% [17]. In our data, we estimated the 24-month HIV-free survival probability to be 78% overall and 82% when the infant dose of NVP was also received. However, due to the sparseness of our data, the confidence intervals around our estimates are very wide and each spans 55%; thus, our data do not allow for any firm conclusions.

At the time the program was implemented, the sdNVP only regimen was offered in Malawi as the predominant option for PMTCT, especially in rural areas. More recently, Malawi-as with other Sub-Sahara African countries-is transitioning to more efficacious regimens. However, even in the context of more efficacious regimens, our analysis to inform HIV free survival remains relevant; a PMTCT program offering more complex ARV regimens still needs to assess the outcomes of the intervention especially when adherence to treatment might decrease with long term and complex regimens.

Moreover, this analysis revealed how a “minimalist” PMTCT approach-sdNVP only antiretroviral regimen-results in a relatively high proportion (around 22%) of HIV-exposed children dead or HIV-infected.

Detection of HIV infections in the study population was essentially based on HIV antibody tests and could not have occurred earlier. A proportion of early deaths could have been avoided if HIV diagnosis were established by 12 months of age and pediatric antiretroviral treatment initiated in a timely manner [3]. If not diagnosed and treated, approximately one-third and one-half of HIV-infected infants will die before the age one year and two years, respectively [18]. Improved coverage, more effective ARV regimens, and early infant diagnosis could have prevented a substantial proportion of these HIV infections and deaths among HIV-exposed children.

This analysis has limitations. First, most of the children, including those who tested HIV-negative, were still exposed to HIV through breastfeeding-99% of children had been breastfed-and this analysis did not adequately capture the definitive HIV status of these HIV-exposed infants.

Second, establishing diagnosis of HIV-infection requires HIV testing at two different points in time, children were tested only at one time [14]. Reasonable conclusions, however, may still be drawn as the HIV test in this circumstance was not meant for diagnosis but for surveillance.

Third, our analysis could not ascertain that all children who were not alive had died of HIV infection; the registers used during home visits did not capture verbal autopsy information.

Next, our analysis likely overestimates the numbers of HIV infected infants. Infants who tested positive for HIV antibodies at ≤ 18 months of age, were considered HIV-infected in this analysis, even though conclusive HIV infection status was unknown.

Finally, this study demonstrates the limitations of analyzing routine service data. Despite efforts to maintain good data quality, routine service data are not comparable to data collected in formal research studies with rigorous protocols for data collection [19]. For example, two-thirds of HIV-exposed children that received ARV prophylaxis could not be included in the analysis because data on their mother’s address were not registered. It is not possible to determine if the rate of HIV-free survival among this group is comparable to those who were followed up through home visits. During program implementation, the practice of documenting the address of mothers was not regularly conducted, which prevented the follow-up of a large proportion of HIV-exposed children and their mothers. This analysis highlights the need to have good quality service data-from both health facilities and home visits. As the quality of service data improves, so does its ability to measure HIV-free child survival rates.

Moreover, among the large proportion of mothers for whom home addresses were not available, there was not a significant difference in their age, parity, or distance between home and the clinic. Despite that there is no existing evidence of bias between the mothers who were LTFU versus those who were tracked through home visits, it cannot be completely ruled out.

Lastly, the study population does not represent a random sample, therefore the results may not be generalizable to a larger population. A greater sample size in a representative number of sites, availability of defined age at the time of HIV testing, as well as documentation of the number and results of previous HIV tests would have allowed more detailed analysis and possibly firmer conclusions.

## Conclusion

Our analysis reports on HIV-free survival among children born to HIV-positive mothers in rural health centers in Malawi. It was possible to couple analysis of routine PMTCT service data with information collected during routine home visits to analyze PMTCT outcomes.

Among our study population, the HIV-free survival rate of HIV-exposed children was between 61% and 82%. However, the confidence intervals around our estimates are quite wide and each span 55%, the natural HIV-free survival rate observed in the absence of any PMTCT intervention. Therefore, this analysis does not lead to a firm conclusion.

In order to better understand-and achieve clear statistical evidence-of HIV-free survival, such service data should be collected routinely, systematically, and in a more representative number of sites. In addition to quantity of data, this analysis highlighted the need to have good quality data from health facilities and home visits, as well as better structured home visits. We recommend that 1) mothers’ addresses are routinely collected to enable follow-up, 2) testing of HIV-exposed infants are done at specific ages (e.g. six weeks, six months, 12 months, 18 months), 3) clinical data are systematically recorded during home visits (duration and type of mother’s ARV regimen and age of HIV testing of children), 4) quality control of data being collected is ensured, and 5) a strategy to drastically reduce LTFU among HIV-positive pregnant mothers is implemented.

## Competing interests

The authors declare that they have no competing interests.

## Authors’ contributions

JM and KT contributed to the conception and design, analysis and interpretation of data; were involved in drafting the manuscript; and gave final approval of the manuscript. TM contributed to the acquisition of data; was involved in drafting the manuscript; and gave final approval of the manuscript. MW made significant contributions to the analysis and interpretation of data; was involved in drafting the manuscript; and gave final approval of the manuscript. CS contributed to the conception and design, analysis and interpretation of data; was involved in revising the manuscript; and gave final approval of the manuscript. RD contributed to the interpretation of data; was involved in drafting and revising the manuscript; and gave final approval of the manuscript. CH contributed to the interpretation of data; was involved in revising the manuscript; and gave final approval of the manuscript. All authors read and approved the final manuscript.

## Pre-publication history

The pre-publication history for this paper can be accessed here:

http://www.biomedcentral.com/1471-2458/12/405/prepub
